# Dynamical systems model of development of the action differentiation in early infancy: a requisite of physical agency

**DOI:** 10.1007/s00422-023-00955-y

**Published:** 2023-01-19

**Authors:** Ryo Fujihira, Gentaro Taga

**Affiliations:** Graduate School of Education, The University of Tokyo, 7-3-1 Hongo, Bunkyo-Ku, Tokyo, 113-0033 Japan

**Keywords:** Infant development, Dynamical systems, Bifurcation, Self-referential, Physical agency, Mobile conjugate reinforcement

## Abstract

Young infants are sensitive to whether their body movements cause subsequent events or not during the interaction with the environment. This ability has been revealed by empirical studies on the reinforcement of limb movements when a string is attached between an infant limb and a mobile toy suspended overhead. A previous study reproduced the experimental observation by modeling both the infant’s limb and a mobile toy as a system of coupled oscillators. The authors then argued that emergence of agency could be explained by a phase transition in the dynamical system: from a weakly coupled state to a state where the both movements of the limb and the toy are highly coordinated. However, what remains unexplained is the following experimental observation: When the limb is connected to the mobile toy by a string, the infant increases the average velocity of the arm’s movement. On the other hand, when the toy is controlled externally, the average arm’s velocity is greatly reduced. Since young infants produce exuberant spontaneous movements even with no external stimuli, the inhibition of motor action to suppress the formation of spurious action-perception coupling should be also a crucial sign for the emergence of agency. Thus, we present a dynamical system model for the development of action differentiation, to move or not to move, in the mobile task. In addition to the pair of limb and mobile oscillators for providing positive feedback for reinforcement in the previous model, bifurcation dynamics are incorporated to enhance or inhibit self-movements in response to detecting contingencies between the limb and mobile movements. The results from computer simulations reproduce experimental observations on the developmental emergence of action differentiation between 2 and 3 months of age in the form of a bifurcation diagram. We infer that the emergence of physical agency entails young infants’ ability not only to enhance a specific action-perception coupling, but also to decouple it and create a new mode of action-perception coupling based on the internal state dynamics with contingency detection between self-generated actions and environmental events.

**Supplementary Information:**

The online version contains supplementary material available at 10.1007/s00422-023-00955-y.

## Introduction

As initial development of human life, physical agency when acting on the environment with the body emerges in infancy (Gergely 2002). While young infants are sensitive to the contingencies between their spontaneous movements and subsequent environmental events, the mechanisms for the organization of action as a physical agency remain unclear (Gergely 2002; Watanabe et al. 2011; Kelso 2016; Sen and Gredebäck 2021; Bednarski et al. 2022). Initial human actions are generated as spontaneous movements. They appear as early as approximately 7.5 weeks of gestational age and are maintained until the early infancy period (Einspieler et al. 2021). It is likely that the initial spontaneous movements are generated in a self-organized fashion and transformed into goal-directed behaviors. Thus, it is crucial to understand the development of such behaviors as a self-organizing process (Thelen et al. 1987; Thelen and Smith 1996). Modeling studies based on a theory of self-organization in dynamical systems have revealed the mechanisms underlying the generation of motor actions, especially rhythmic movements (Haken et al. 1985; Taga et al. 1991; Kelso 2021; Taga 2021). However, attempts to construct a theoretical model for the development of infant actions remain limited. Kelso and Fuchs (2016) presented a dynamical system model for reproducing infant behaviors during mobile conjugate reinforcement (MCR) as introduced by Rovee and Rovee (1969). In the MCR paradigm, a string was attached between the infant’s leg and a mobile toy suspended above the infant. Infants quickly “realized” that their spontaneous leg movements caused the mobile to move and increased the amount of movement. Kelso and Fuchs (2016) reproduced such infants’ learning phenomena as positive feedback loop between the nonlinear oscillators representing the limb and mobile movement. In the present study, we extended the model of Kelso and Fuchs (2016) to reproduce wider experimental observations in relation to the development of infant behavior as a physical agency.

A large number of empirical studies on young infants have shown that they can change actions based on reinforcement stimuli. Newborn infants exhibit evidence of reinforcement in non-nutritive sucking with the mother’s voice (DeCasper and Fifer 1980), arm movements with facial images of mothers (Walton et al. 1992), and arm movements with the live video of their own arm movements (Van Der Meer et al. 1995). The most widely used experimental method for infants is MCR (Rovee and Rovee 1969). The MCR has been used to test infants’ learning and memory; moreover, detailed analysis of the motor actions with the whole body during MCR has provided further clues to understanding the mechanism for organizing actions. The increase in movement occurred particularly at the limb to which the string was attached (Rovee-Collier et al. 1978; Watanabe and Taga 2006, 2009; Jacquey et al. 2020b). Infants can also learn a specific pattern of intra-limb coordination, such as knee and hip extension angles (Angulo-Kinzler 2001) and ankle raising heights (Sargent et al. 2014). These results indicate that movement enhancement is not just an exhibition of joyfulness, but also the result of learning sensorimotor coordination through dynamic interactions with the environment.

**Fig. 1:**
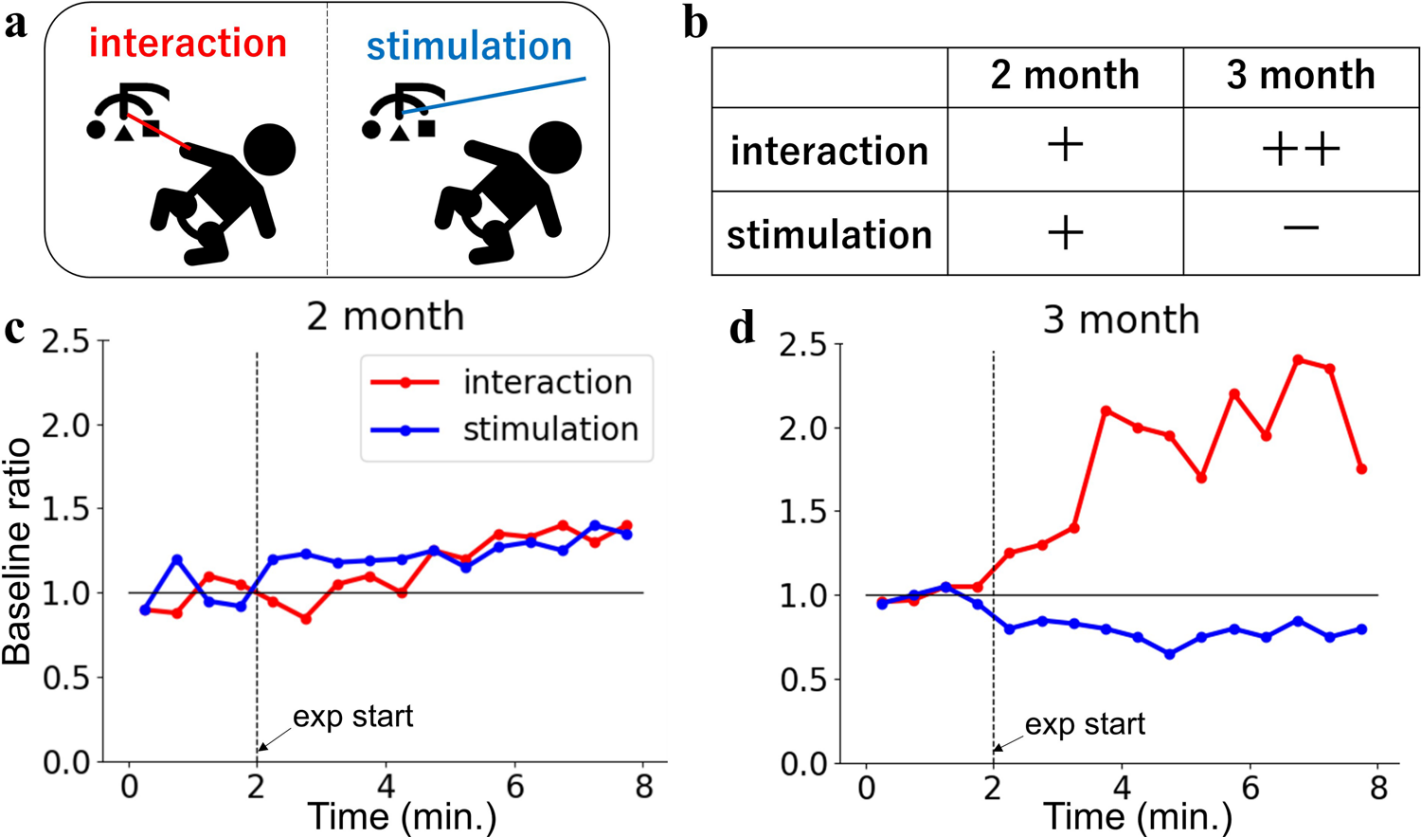
Experimental results of Watanabe et al. (2011). The degree of limb movement was measured as a change in baseline ratio, which was defined as a ratio of an average arm’s velocity over successive 30-s periods to that of first 2-min baseline. **a** An illustration of two conditions of this experiment; infants moved the mobile in interaction condition and other people moved the mobile in stimulation condition. **b** Two-month-old infants increased their limb movement (+) in both conditions. Three-month-old infants largely increased their limb movement (++) in the interaction condition and decreased it (−) in the stimulation condition. **c**, **d** Actual experimental results extracted from Figs. 4 and 5 in Watanabe et al. 2011. The experimental condition began at 2 min as shown on the *x*-axis, when the mobile began to be moved

In addition to the ability to enhance actions based on reinforcement information from the environment, infants are likely to detect the presence or absence of causality between self-generated actions and environmental changes, and to inhibit unnecessary actions. Rochat and Striano (1999) used a mechanical pacifier linked to sound generation and confirmed an increase in non-nutritive sucking with a contingent sound and a decrease with a noncontingent sound in 2-month-old infants. Lewis et al. (1985) provided infants with audio-visual stimuli contingent or noncontingent on arm movements, and found that arm movements decreased for noncontingent events. Heathcock et al. (2004) used the MCR paradigm to examine the differences in infants’ actions toward a mobile under two conditions, i.e., where the mobile was moved by the infants themselves or by others. Watanabe et al. (2011) revealed the development of action differentiation using the MCR paradigm. In particular, 3-month-old infants increased their arm movements if the mobile was moved by the infants themselves (called “interaction condition”), and decreased their arm movements if the mobile was moved by others (called “stimulation condition”), as shown in Fig. 1a, b. In contrast, the actions of 2-month-old infants were not differentiated between the two conditions. The time course of the arm movements is shown in Fig. 1c, d. These results indicate that infants develop the ability to differentiate actions based on the circular causality of their spontaneous movements and consecutive environmental events between 2 and 3 months of age.

This contingency detection ability requires a self-referential mechanism regarding feedforward copy of the commands to muscles referred to as “efference copy” (von Holst and Mittelstaedt 1950) or a feedforward of the expected sensory consequences referred to as “corollary discharge” (Sommer and Wurtz 2008; Sperry 1950). Such a mechanism has been discussed in terms of physical agency or self-awareness (Gallagher 2000; Blakemore and Frith 2003). In particular, the first 3 months after birth are critically important for the development of physical agency (Gergely and Watson 1999; Jacquey et al. 2020a). In recent years, the neural responses of 3-month-old infants related to mobile contingencies have been investigated using electroencephalography (EEG) (Zaadnoordijk et al 2020; Meyer and Hunnius 2021). Zaadnoordijk et al. (2020) suggested that the mismatch negativity of EEG with the disconnection of a contingent mobile is evidence of physical agency, because it reflects the infants’ expectations regarding the consequences of their actions on the mobile. Meyer and Hunnius (2021) also showed that the event-related potential of the EEG differs between self-produced events and external irregular events. These results imply a neural substrate for the detection of causal links between self-generated actions and the consequences of environmental changes in 3-month-old infants, although the development of such a mechanism is not clear.

To clarify the mechanisms underlying infant behavioral changes during the MCR, Zaadnoordijk et al. (2018) introduced a reinforcement learning model with discretized states for the limb and mobile and reinforcement stimuli that induced the increase of limb movements. While the model simply incorporated an automatic process to reinforce limb movements contingent to the mobile movements without creating the representation of causality between self-movements and mobile movements, it replicated the enhancement of limb movements connected to the mobile, which were observed in Rovee-Collier et al (1978). However, the model could not exhibit a sharp increase of limb movement immediately after the mobile disconnection (Rovee-Collier et al. 1978), which inferred the presence of sense of agency. Zaadnoordijk et al. (2018) claimed that incorporating additional mechanisms is needed to model the agentic behavior. On the other hand, Kelso and Fuchs (2016) argued that the sensory motor coordination is a basis for the emergence of agency and dynamical systems approach is a promising avenue to tackle with this issue. They proposed a coupled oscillator model for a limb and mobile with positive feedback for the MCR. The model successfully reproduced experimental observations of the time courses of kick responses in the MCR (Rovee and Rovee 1969). The simulation results also revealed that a bifurcation from a weakly coupled state to a highly coordinated state between the infant and mobile movements is a key mechanism underlying the behavioral changes. Kelso (2016) further argued that a sudden and sustained amplifying effect on the actions from the positive feedback induces the discovery of oneself as a causal agent. However, no theoretical study has aimed to reproduce the aforementioned empirical observations on the differentiation of actions, as shown in Fig. 1 (Watanabe et al. 2011). A crucial point is that infants exhibit not only enhancements of actions but also inhibitions of actions depending on the causal link between the action and environmental events. Thus, in this study, we extend the dynamical systems model of Kelso and Fuchs (2016) by incorporating the reference of self-movements and dynamics creating a bifurcation between the enhancement and inhibition of actions. In the present study, we reproduce the experimental observations of Watanabe et al. (2011) by constructing a dynamical systems model including the internal dynamics of the developing brain and discuss the developmental origins of physical agency.

## Methods

**Fig. 2:**
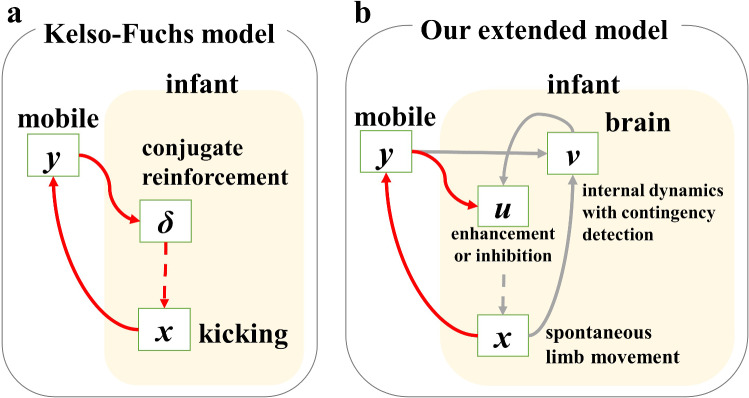
Conceptual diagram of the model’s improvement. **a** Kelso–Fuchs model. **b** Our extended model. Each character written in rectangles is the variables of the model. A new variable *v* is governed by internal dynamics with input of detected contingency between spontaneous limb movements *x* and mobile movements *y*. *u* in the extended model is equivalent to *δ* in Kelso–Fuchs model. *u* receives additional input from *v* and enhances or inhibits spontaneous limb movements. We postulate that *u* and *v* represent the state of the brainstem and cerebral cortex, respectively. *x* is a collective variable to be generated by the interaction among the CPG in the spinal cord and the limb. The arrows in diagrams mean the interaction between variables. Red arrows show that the effects are always positive and gray arrows show that the effects are changeable. Solid arrows represent change in value, and dotted arrows represent frequency modulation. The equations are written in the method section

A dynamical systems model of the MCR paradigm (Kelso and Fuchs 2016) formulated the infant limb movements as a nonlinear limit cycle oscillator, movements of the mobile as a linear damped oscillator, and functional coupling between the two, as shown in Fig. 2a.

The model is referred to as the Kelso–Fuchs (KF) model and consists of three equations as follows:

(Equation of Kelso–Fuchs model)

1 $$ \ddot{x} + \dot{x}\left\{ {\gamma + \alpha x^{2} } \right\} + x\left\{ {\omega_{0}^{2} + \delta x^{2} } \right\} = 0 $$

2 $$ \ddot{y} + \varepsilon \dot{y} + \Omega_{0}^{2} y = cx $$

3 $$ \dot{\delta } = ay^{2} - \kappa \delta $$

Equation 1 is a van-der-Pol oscillator with an additional Duffing term, where *x*(*t*) represents the infant limb movements. (*γ* + *αx*^2^) is a nonlinear damping term and leads to a stable limit cycle when *γ* < 0 and *α* > 0. This is based on the experimental observations showing that the amplitude of infant’s kicks stays relatively constant and only the kick frequency changes. The oscillation frequency is determined by $\omega_{0}$ and the additional Duffing term *δx*^2^. *δ*(*t*), a variable in Eq. 3, has a positive feedback effect on the limb oscillation frequency. Equation 2 is a damped harmonic oscillator, where *y*(*t*) represents the mobile movement. *ε* is a damping term and $\Omega_{0}$ determines the natural oscillation frequency. *c* determines the connection between the infant and mobile. If *c* = 0, the mobile oscillator is disconnected from the infant limb oscillator. If *c* is larger than 0, the mobile oscillator is forced by the infant oscillator. Equation 3 expresses the interaction between *x*(*t*) and *y*(*t*). The movement of the mobile oscillator *y*(*t*) triggers an increase in *δ*(*t*), leading to an increase in the frequency of the limb oscillator. *a* is a key parameter for determining the strength of the positive feedback regarding the activation of the mobile movement. *κ* is an exponential decay parameter for limiting the maximum increase when *x* and *y* are coupled, and inducing decay when decoupled.

The KF model replicates the experimental results of the MCR proposed by Rovee and Rovee (1969). The key mechanism in the KF model is positive feedback, which induces a phase transition of the infant and mobile from an uncoupled state to a coupled state. Based on the results of the KF model, Kelso (2016) argued that this phase transition is a manifestation of the emergence of physical agency. However, the KF model was not designed to replicate the experimental results regarding action differentiation (Watanabe et al. 2011) as shown in Fig. 1, a requisite for the emergence of physical agency.

Thus, we extended the KF model to include a new variable *v*(*t*) representing the brain function involved in action differentiation, as shown in Fig. 2b. The dynamics of *v* has the capacity to switch from one state to another to change actions. *u*(*t*) in our model is equivalent to *δ*(*t*) in the KF model and regulates the oscillation frequency of *x*(*t*). Note that an arm was connected to the mobile in the experiment (Watanabe et al. 2011). The arm movements are generally complex as compared with kicking movements of the leg, and quantifying movement changes in the MCR paradigm is not a simple task. Since the limb movements were continuously generated throughout the experiment in the analyzed infants, the measured velocities of limb movements were averaged for a certain period to reveal the changes in amount of movements for both of the arm and the leg regardless the activity is periodic or aperiodic. In the present model, we assumed that rhythmic activity with changing amplitude and frequency is a minimum description of the continuous changes in such complex movements of the arm. Thus, the arm movements *x* were governed by the van-der-Pol oscillator with frequency modulation. Here we postulate that *x*(*t*) represents a collective variable that is generated through the interaction of the CPG and the limb. We also postulate that *u*(*t*) represents the brainstem involved in the frequency control of the central pattern generator (CPG) for rhythmic movements of the limbs (Grillner 1985), and *v*(*t*) represents the cerebral cortex involved in the selection of actions based on the audio-visual sensory signals induced by the mobile movements and signals of self-movement. The arrow from *y* to *v* represents the sensory signals, and the arrow from *x* to *v* represents the signals of self-movement. *v*(*t*) receives the sensory signals and the signals of self-movement, detects the congruence between them, and thereby regulates *u*(*t*). The equations of the model are as follows:

(Equation of our extended model)

4 $$ \ddot{x} + \dot{x}\left\{ {\gamma + \alpha x^{2} } \right\} + x\left\{ {\omega_{0}^{2} + u} \right\} = 0 $$

5 $$ \ddot{y} + \varepsilon \dot{y} + \Omega_{0}^{2} y = cx + f $$

6 $$ \tau_{1} \dot{u} = ay^{2} + vy^{2} - u $$

7 $$ \tau_{2} \dot{v} = x^{2} y^{2} - y^{2} + bv - v^{3} $$

Equations 4, 5, and 6 are based on Eqs. 1, 2, and 3, respectively. In Eq. 4, the van-der-Pol oscillator for the limb movements originally had an additional Duffing term *δx*^2^ in the KF model; in the present model, it was changed to *u* without multiplying by *x*^2^ to simplify the model. In Eq. 5, i.e., the damped harmonic oscillator for the mobile movements, we added the input of external force *f*, a sine wave with a constant amplitude and constant frequency. This was used to examine the stimulation condition, in which an experimenter moved the mobile to replicate the movement of the mobile produced by the infant under the interaction condition (Watanabe et al. 2011). Equation 6 determines the changes in *u*, which increases or decreases the oscillation frequency of the limb movements. In contrast to Eq. 3, *u* evolves into a positive or negative value depending on the additional input *vy*^2^. Equation 7 was also incorporated into the model. Whether the time evolution of *v* shows a positive or negative value is determined by the input and internal dynamics. The input term *x*^2^*y*^2 ^*− y*^2^ provides a positive effect if *x* and *y* oscillate synchronously, and a negative effect if *x* and *y* oscillate asynchronously. This indicates that the relationship between signals of self-movement and sensory signals affects the time evolution of *v.* The internal dynamics take the form of *bv − v*^3^, creating a bifurcation generally referred to as a supercritical pitchfork bifurcation (Strogatz 2018). The variable *v* is monostable if *b* < 0 (the fixed point is *v* = 0) and bistable if *b* > 0 (the fixed points are *v* = $\pm \sqrt{b}$). Here, we assumed that parameter *b* is a control parameter for producing age-dependent differences in the action differentiation. Lastly, we introduced the time constants *τ*_1_ and *τ*_2_ for *u*(*t*) and *v*(*t*), respectively. We defined *τ*_2_ as larger than *τ*_1_, so *v*(*t*) evolved slower than *u*(*t*).

For the simulation, the following parameters were used: *γ* = − 0.25, *α* = 1, *ε* = 0.5, $\omega_{0}$ = 0.6, $\Omega_{0}$ = 2.2, $\tau_{1}$ = 10, $\tau_{2}$ = 200, and *a* = 3. In accordance with the experimental observations shown in Fig. 1, we simulated the baseline period for the first 200 s (*c* = 0 and *f* = 0) and experimental period for the following 800 s in either the interaction condition (*c* = 2 and *f* = 0) or stimulation condition (*c* = 0 and *f* = 2sin(2*πt*/6)). The simulations were repeatedly performed under various values of the control parameter *b* between − 10 and 20. The differential equations for the model were integrated numerically with a time step of 0.1 using the Odeint Scipy library for Python, which solves ordinary differential equations using lsoda from the FORTRAN library odepack. In general, it uses Adams methods (predictor–corrector) for nonstiff cases and backward differentiation formula methods (gear methods) for stiff cases.

## Results

**Fig. 3:**
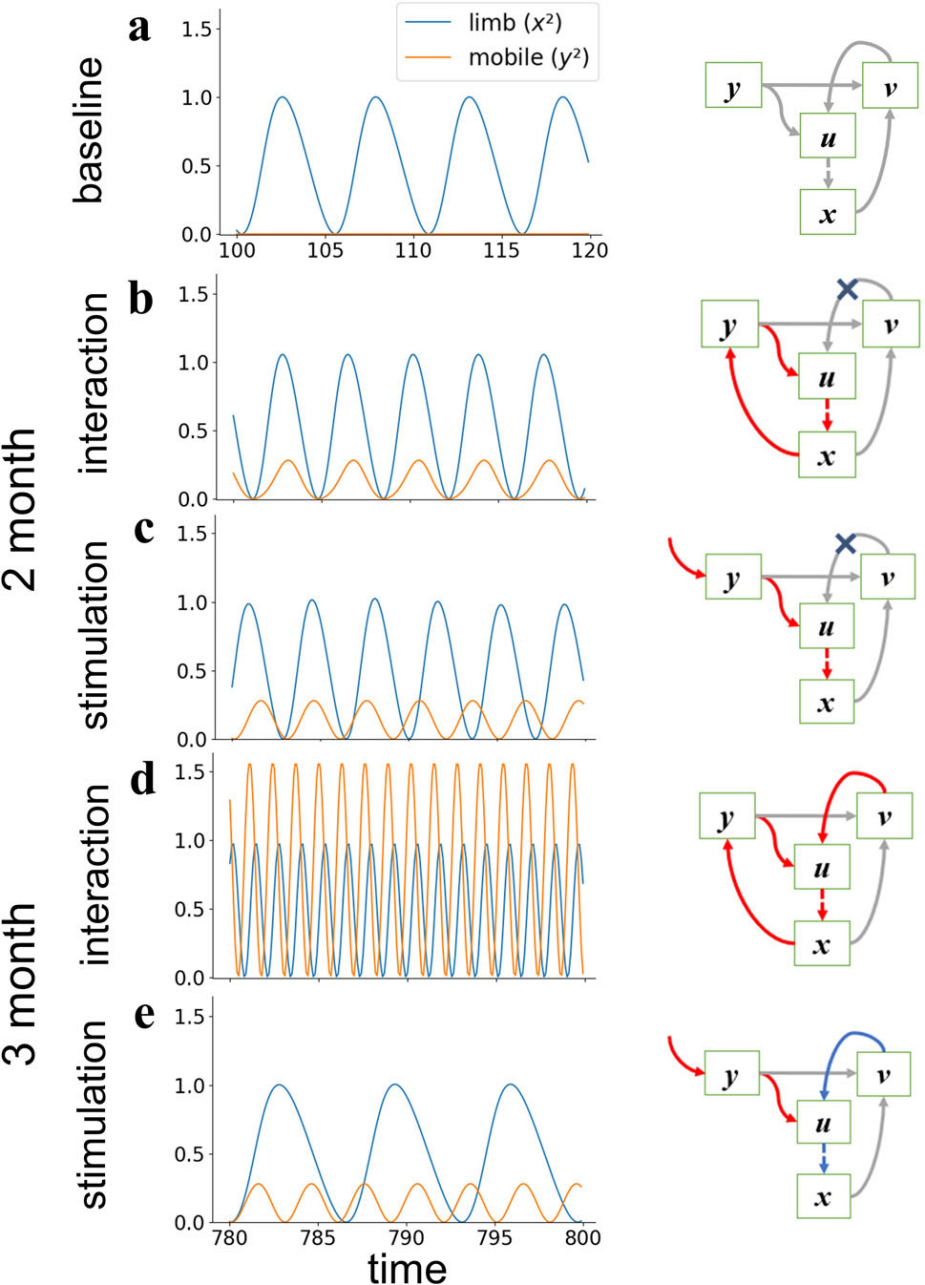
Limb and mobile oscillations in the steady-state and conceptual diagrams of the connection of variables in each age and condition arranged from Fig. 2b. Red and blue arrows in the diagrams show positive and negative effects, respectively. Additional cross on arrows means that the connection does not work. **a** Baseline (*c* = 0, *f* = 0). There is no interaction between variables. **b** Interaction condition of 2-month-old (*b* = − 5, *c* = 2, *f* = 0). When *v* = 0 is stable, the connection from *v* to *u* does not work. **c** Stimulation condition of 2-month-old (*b* = − 5, *c* = 0, *f* = 2sin(2*πt*/6)). **d** Interaction condition of 3-month-old (*b* = 15, *c* = 2, *f* = 0). *v* takes a positive value and provides a positive input to *u*. **e** Stimulation condition of 3-month-old (*b* = 15, *c* = 0, *f* = 2sin(2*πt*/6)). *v* takes a negative value and provides a negative input to *u*. See also Supplementary Video 1–5

We simulated the experimental phase in the interaction and stimulation conditions under various values of the control parameter *b*. We found that the results with *b* = − 5 and 15 reproduced the observations for 2- and 3-month-old infants, respectively, and were labeled as such thereafter. Figure 3 illustrates the steady-state dynamics of the limb and mobile in each conditions for each age. Figure 3a shows the steady-state dynamics in baseline, which is common for all conditions and ages. The limb oscillates as a pure van-del-Pol oscillator in the baseline, when there is no input to the mobile device (*c* = 0, *f* = 0).

Figure 3b–e shows the steady-state dynamics in the experimental phase. While the oscillation frequency of a 2-month-old is similar between the different conditions (Fig. 3b, c), that of the 3-month-old is dramatically different between the different conditions (Fig. 3d, e). In addition, the mobile and limb oscillate synchronously in the interaction condition (Fig. 3b, d) and asynchronously in the stimulation condition (Fig. 3c, e).

To compare the results from the simulations with experimental observations, we calculated the baseline ratio, i.e., the ratio of the mean frequency of the limb oscillation over successive 100 s periods to the mean oscillation frequency of the first 200 s (i.e., the baseline). The mean oscillation frequency was obtained simply by counting the number of cycles in each period.

**Fig. 4:**
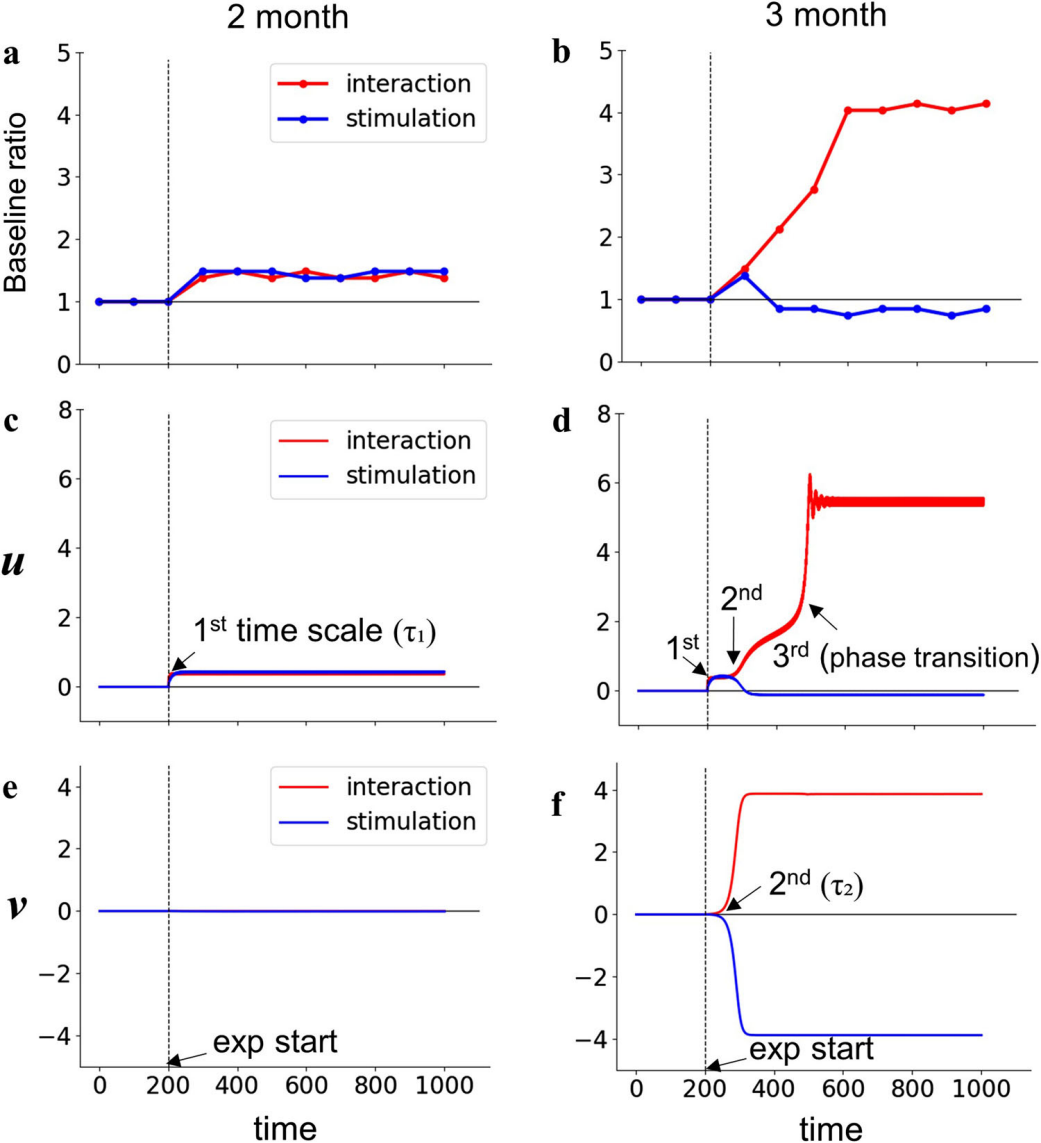
**a**, **b** Simulated time evolution of the baseline ratio. We calculated a mean frequency of the limb oscillation over successive 100-s periods and took the ratio to the mean oscillation frequency of the first 200-s baseline as a baseline ratio. They are the replication of Fig. 1c, d of the results of Watanabe et al. (2011). **c**, **d** Time series of *u*; the variation is qualitatively equivalent to that of the baseline ratio. **e**, **f** Time series of *v*. In each figure, the red line is the simulated result of the interaction condition (*c* = 2, *f* = 0) and the green line is the simulated result of the stimulation condition (*c* = 0, *f* = 2sin(2*πt*/6))

Figure 4a, b shows the entire time course of the baseline ratio for each condition for each age. The time course is in good agreement with the experimental results: 2-month-old infants show enhancements of movements in both conditions, and 3-month-old infants show enhancement in the interaction condition and inhibition in the stimulation condition (Fig. 1c, d). Figure 4c, d shows the entire time course of *u*(*t*) for the corresponding conditions and ages. The change in *u*(*t*) is qualitatively similar to that in the baseline ratio, because *u*(*t*) directly affects the limb oscillation frequency. Figure 4e, f shows the entire time course of *v*(*t*) for the corresponding conditions and ages. While the value of *v*(*t*) converges to a monostable state in both conditions at 2 months of age, it converges to either positive or negative values depending on the conditions at 3 months of age.

**Fig. 5:**
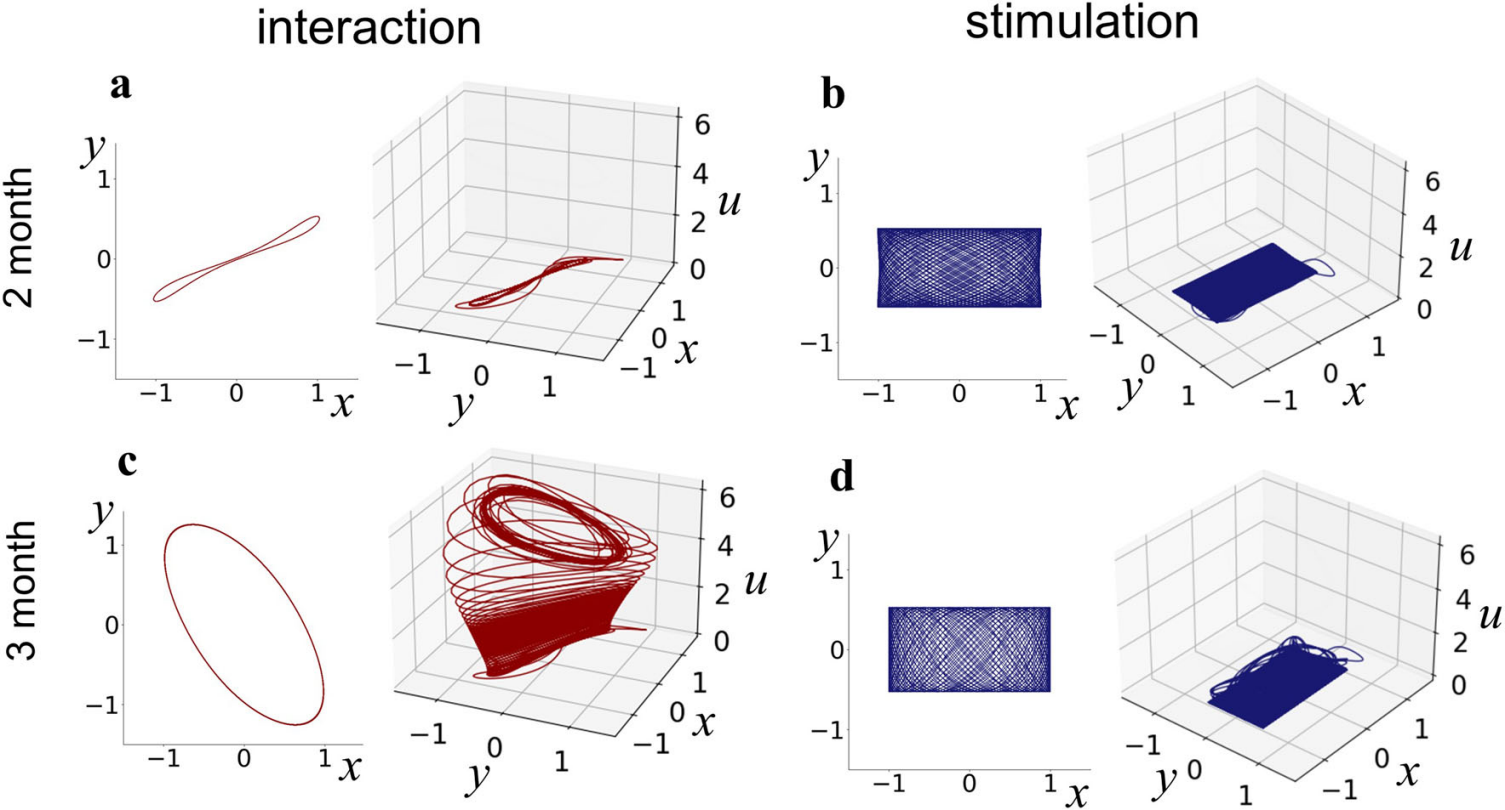
Steady-state trajectories in *x*–*y* plane and whole-time trajectories in *x–y–u* space. *x* and *y* are synchronized in the interaction condition and not synchronized in the stimulation condition. **a** Interaction condition of 2-month-old. **b** Stimulation condition of 2-month-old. **c** Interaction condition of 3-month-old. **d** Stimulation condition of 3-month-old. Also see Supplementary Video 2–5

Examining the time courses of *u*(*t*) and *v*(*t*) at 3 months of age in detail, it turns out that they evolve in a specific sequence, as shown in Fig. 4d, f. Immediately after the beginning of the experimental phase, the movements of the mobile induce a positive input *ay*^2^, and the variable *u* shows a subtle increase over the timescale determined by $\tau_{1}$ in both the interaction and stimulation conditions. The variable *v*(*t*) evolves over the timescale of $\tau_{2}$ and converges to a positive value in the interaction condition and negative value in the stimulation condition. The changes in *v*(*t*) further induce the evolution of *u*(*t*) toward a positive value in the interaction condition and negative value in the stimulation condition. Finally, the positive value of *u*(*t*) in the interaction condition exhibits a transition to a larger positive value emerging from the interaction between the limb *x*(*t*) and mobile *y*(*t*). This phase transition was also observed in the KF model (Kelso and Fuchs 2016). Notably, such a change occurs only under the interaction condition. Figure 5 shows steady-state trajectories of the movements of the limb *x*(*t*) vs those of the mobile *y*(*t*) in the *x*–*y* plane, and whole-time trajectories of *x* versus *y* versus *u*.

At 2 months of age, *x*(*t*) and *y*(*t*) show in-phase synchronization in the interaction condition (Fig. 5a), whereas they show asynchronous movements in the stimulation condition (Fig. 5b). At 3 months of age, *x*(*t*) and *y*(*t*) initially show in-phase synchronization and, as *u*(*t*) grows, they show a transition to an anti-phase synchronization state in the interaction condition (Fig. 5c). Under the stimulation condition, asynchronous movements are observed (Fig. 5d). Supplementary Videos 2–5 provide animations of the whole simulation results.

**Fig. 6:**
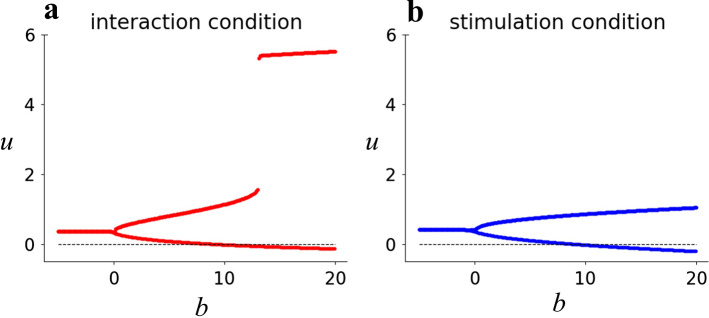
Bifurcation diagrams: The fixed point for *u* as a function of *b*. **a** Fixed point calculated with c = 2, *f* = 0 (interaction condition). **b** Fixed point calculated with *c* = 0, *f* = 2sin(2*πt*/6) (stimulation condition)

The difference in actions between 2-month-old and 3-month-old infants was reproduced only by the different values of *b*. We obtained a bifurcation diagram of *u* as a function of *b* for each interaction and stimulation condition (Fig. 6).

In the parameter region of *b* < 0, *u*(*t*) converges to a single fixed point. We have already demonstrated the result with *b* = − 5, i.e., it reproduces only the enhancement action of the 2-month-old infants. An increase in the value of *b* yields bifurcation into bistable fixed points at *b* = 0, primarily caused by the incorporated dynamics of *v*(*t*). A further increase in the value of *b* exceeding 12 yields a transition from one of the fixed points to a new fixed point with a higher value, corresponding to the transition reported in the KF model (Kelso and Fuchs 2016). This bifurcation occurs only under the interaction condition. We demonstrated the result with *b* = 15 as a model to reproduce the differentiated actions depending on the conditions in 3-month-old infants. Despite the presence of two fixed points, the solution starting from the initial condition (*u* = 0 and *v* = 0) converges to one fixed point depending on the conditions. This property is induced by the input value of *x*^2^*y*^2 ^*− y*^2^ to the changes in *v* in Eq. 7. As the term *x*^2^*y*^2 ^*− y*^2^ takes a positive value in the interaction condition, it induces the evolution of *v* to the positive fixed point. In contrast, as the term *x*^2^*y*^2 ^*− y*^2^ takes a negative value in the stimulation condition, it induces the evolution of *v* to the negative fixed point.

**Fig. 7:**
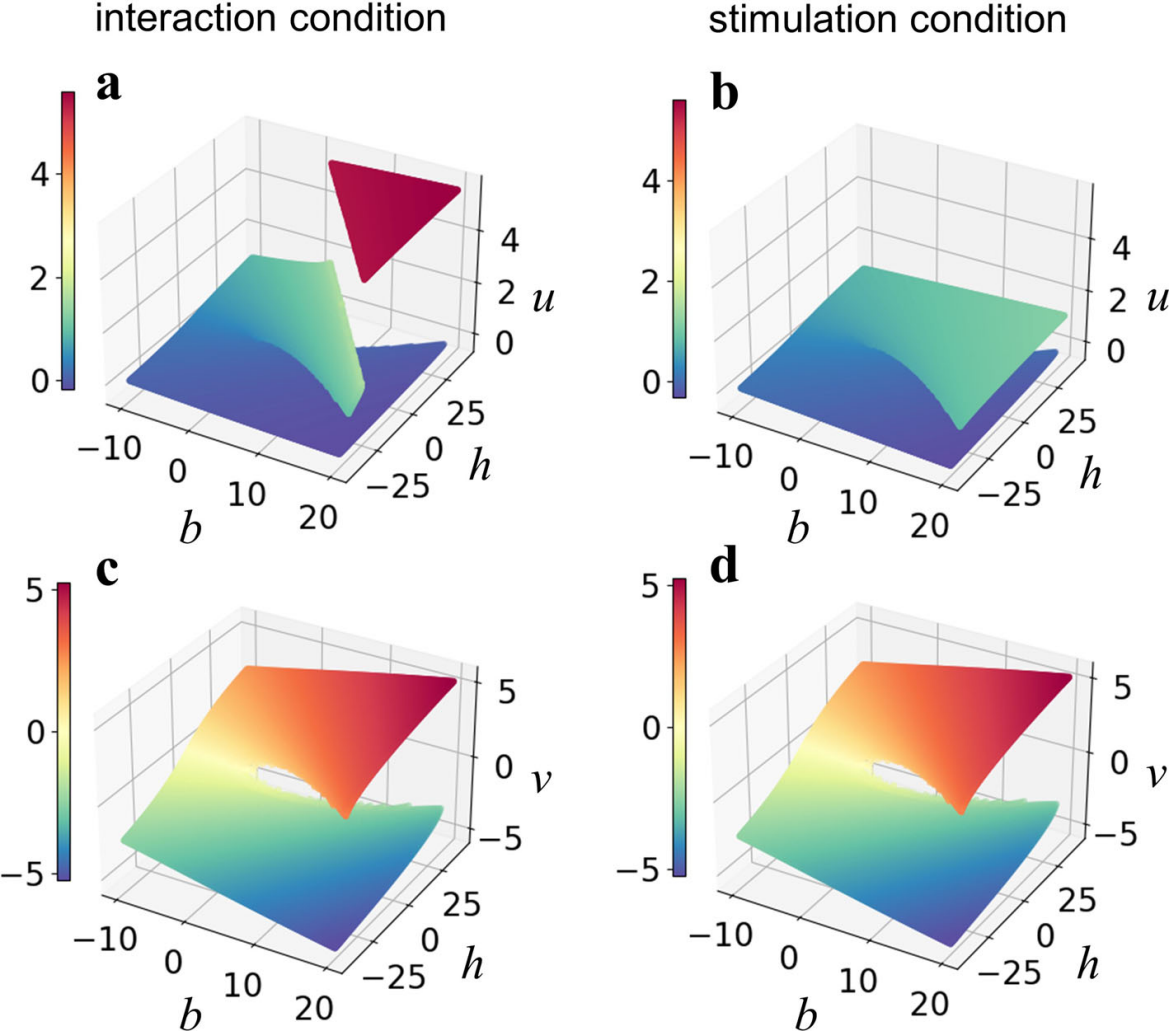
Three-dimensional bifurcation diagram: **a**, **b** show the fixed point for *u* as a function of *b* and *h*. **c**, **d** show the fixed point for *v* as a function of *b* and *h*. In **a**, **c**, the fixed point was calculated with *c* = 2, *f* = 0 (interaction condition). In **b**, **d**, the fixed point was calculated with *c* = 0, *f* = 2sin(2*πt*/6) (stimulation condition)

We also replaced the term *x*^2^*y*^2 ^*− y*^2^ with a new parameter *h* and calculated a three-dimensional bifurcation diagram of *u* as a function of *b* and *h*. This manipulation enabled us to examine the parameter dependence of the behavior of the model without any fluctuations. Figure 7 shows the fixed point surface, the shape of which is known as a cusp catastrophe, i.e., an elemental catastrophe (Thom 1974).

Consistent with Fig. 6, a surface with higher values of *u* only emerges under the interaction condition (Fig. 7a), and it emerges as a function of both *b* and *h*.

Finally, we examined the memory capability after the experimental periods. Although Watanabe et al. (2011) did not conduct the operation of mobile disconnection, it has been widely implemented in the MCR paradigm to test memory (e.g., Rovee-Collier et al. 1980; Watanabe and Taga 2006). The increased limb activity observed during the extinction was also associated with infants’ attempt to re-establish the lost relationship (Rovee-Collier et al. 1978). Furthermore, the stimulation after the experience of the first mobile experiment, called “reactivation,” was associated with a mechanism by which early experience can continue to influence behavior for a long term (Rovee-Collier et al. 1980).

**Fig. 8:**
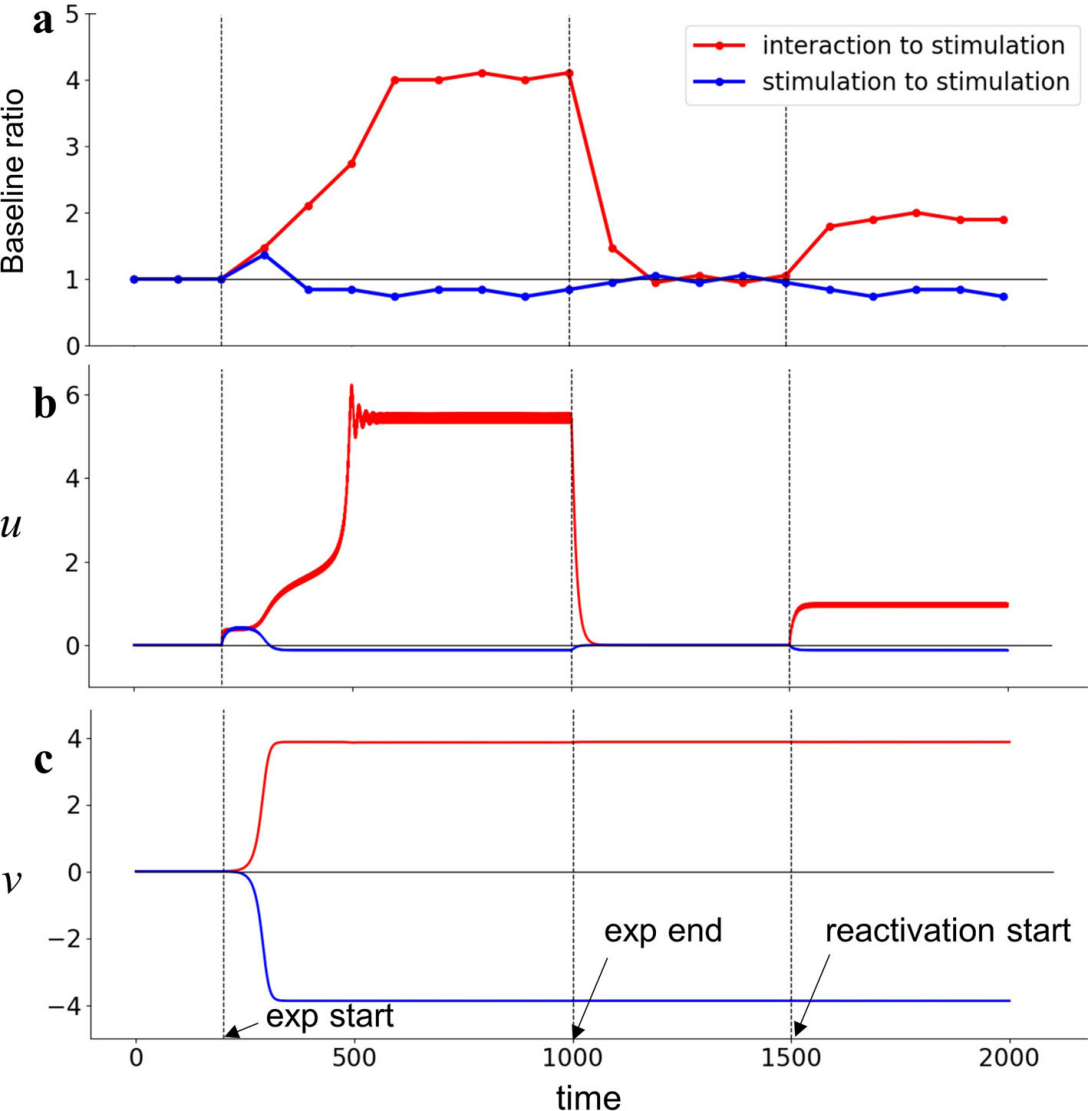
Time evolutions of **a** baseline ratio, **b** *u*(*t*) and **c** *v*(*t*) of the model of 3-month-old (*b* = 15) including memory dynamics. The simulation consists of four phases: baseline (*c* = 0, *f* = 0; *t* = 0 to 200), experimental phase (*c* = 2, *f* = 0 in interaction condition, and *c* = 0, *f* = 2sin(2*πt*/6) in stimulation condition; *t* = 200 to 1000), extinction phase (*c* = 0, *f* = 0; *t* = 1000 to 1500) and reactivation phase (*c* = 0, *f* = 2sin(2*πt*/6); *t* = 1500 to 2000). Red lines are the results whose experimental phase was interaction condition, and blue lines are the results whose experimental phase was stimulation condition

We conducted simulations for baseline, experimental phase, extinction phase and reactivation phase. Figure 8 shows the obtained time courses of baseline ratio, *u*(*t*) and *v*(*t*) with *b* = 15 representing 3-month-old infants. In the experimental phase, either interaction or stimulation condition was examined. The time evolution in baseline condition (*t* = 0 to 200) and experimental conditions (*t* = 200 to 1000) in Fig. 8 is the same as the one in Fig. 4b, d, f: convergence to a positive value in the interaction condition and to a negative value in the stimulation condition. Then, in extinction phase (*t* = 1000 to 1500), we removed the input to the mobile (*c* = 0, *f* = 0). In extinction after the interaction, the baseline ratio immediately decreased due to the immediate decrease in *u*(*t*) toward zero. This shows that the model could not replicate the sharp increase of limb movement immediately after the disconnection (Rovee-Collier et al. 1978). In reactivation phase (*t* = 1500 to 2000), the model produced different behaviors depending on the conditions of the experimental phase: The limb movements increased after experiencing interaction condition and decreased after experiencing stimulation condition. This was caused by the memory property of *v*(*t*) maintaining a positive value after interaction condition and a negative value after stimulation condition. This indicates that *v*(*t*) functions as a memory for the experimental conditions and influences the later behavior.

## Discussion

### Developmental dynamics for action differentiation

In this study, we developed a dynamical system model for explaining action differentiation depending on the relationship between the infant and environment in MCR. In addition to a pair of limb and mobile oscillators with positive feedback for reinforcement as introduced by the KF model (Kelso and Fuchs 2016), bifurcation dynamics were incorporated for selecting the enhancement or inhibition of self-movements in response to detecting contingencies between the limb and mobile movements. Whereas the monostable state led to an inability to differentiate actions, the bistable state made it possible to differentiate actions. The changes in the control parameter *b* induced bifurcation between the monostable (*b* = − 5) and bistable (*b* = 15) states; this was in good agreement with the behaviors of the 2- and 3-month-old infants in Watanabe's experimental study, respectively (Watanabe et al. 2011).

This study showed that the time evolutions of the variables in this model were qualitatively different for different values of the control parameter and under different experimental conditions. In the 2-month parameter regime, the variable *u* was increased in response to the onset of the mobile oscillation over the timescale determined by *τ*_1_; correspondingly, the limb oscillation frequency was slightly increased in both of the interaction and stimulation conditions. In the 3-month parameter regime, the variables of the system evolved over three timescales. First, the time evolution of *u* over the timescale determined by *τ*_1_ was similar to that in the 2-month regime. Then, the variable *v*(*t*) evolved over the second timescale determined by *τ*_2_ toward a positive value in the interaction condition and a negative value in the stimulation condition. These changes in *v* further induced changes in *u*, leading to the enhancement or inhibition of the limb oscillations. Third, the phase relationship between the *x* and *y* oscillations changed from in-phase to anti-phase synchronization and further enhanced the limb oscillation only in the interaction condition. Taken together, the present model not only reproduced the specific results of previous experimental studies, but also provided predictions to be tested in future studies. For instance, the time evolution of the limb movements over the first and second timescales in the interaction condition with 3-month-old infants was not evident in the infant-averaged curve for the baseline ratio in the experiment (Watanabe et al. 2011). To clarify this, a detailed analysis of this behavior is required on an individual basis. The emergence of the phase transition toward the further enhanced state over the third timescale in the interaction condition with 3-month-old infants has not been proven in experimental studies. Future studies should test the aforementioned predictions of the model by analyzing behavioral data on an individual basis.

### Internal state dynamics and memory

The dynamic changes in variables *u* and *v* in this model can be regarded as those of the internal state of the infant brain for producing actions over multiple timescales. Such dynamics were also detailed in previous modeling studies on the dynamical systems of infant behaviors, such as studies on habituation and dishabituation (Schöner and Thelen 2006) and the A-not-B error effect (Aerdker et al. 2022). While behavioral patterns and forms of interaction with the environment in the MCR tasks are different from those in the habituation and dishabituation tasks and A-not-B tasks, the timescale for behavioral changes in these tasks are comparable. Thus, it is important to study the commonalities and differences in the internal state dynamics among the behaviors of infants in future studies.

The MCR paradigm was originally discussed in relation to infants’ long-term memories (Rovee-Collier and Sullivan 1980; Watanabe and Taga 2006; Sen and Gredebäck 2021). In our model, the variable *v* functions as a memory for the experimental conditions. For the simulation of 3-month-old infants, *u* declines immediately after the disconnection of the mobile, whereas *v* maintains the value of the experimental periods, i.e., a positive value in the interaction condition and negative value in the stimulation condition. Although these results demonstrate the mechanisms of the memory generation and maintenance, the model did not include a decay property for the memory. Thus, the model in the present study cannot account for amnesia in infants. Further extension of the model to incorporate long-term memory dynamics is crucial for future studies. We further performed a simulation of limb movement changes when exposed to a series of conditions such as baseline, interaction or stimulation condition, extinction, and stimulation conditions. Since the dynamics of the present model had bistable attractors, the generated action was not solely determined by the environmental condition but showed a hysteresis effect. Thus, the model predicts that a different pattern of actions is generated even in the same environmental condition, if the brain had memory for the experience of the action-perception cycle.

### Putative neural mechanisms

In our model, the bifurcation and phase transitions were induced by increases in the parameter *b*. What is the neural substrate for the parameter *b*? Watanabe et al. (2011) argued that the change from 2-month-old to 3-month-old behavior in MCR could be explained based on changes from subcortical to cerebral cortex-based mechanisms. In addition, recent progress in studies of the subplate (Molnár et al. 2020) suggests that the subplates may be involved in developmental changes in behavior. The subplate is formed prior to the cortical plate in the early fetal period and then plays an important role in establishing the cortical layer (Ohtaka-Maruyama et al. 2018), thalamocortical tract (Molnár et al. 2020) and cortico-cortical network (Kostović 2020) in later fetal periods. Around the term period, it degenerates owing to apoptosis, and the cortical layers become a major part of the cortex (Molnár et al. 2020). Thus, the putative neural mechanism for the behavioral changes in MCR is discussed in relation to the model herein.

Spontaneous movements appear early in the fetal period and are likely induced by the spinal cord (de Vries et al. 1982). As the subplate develops, the spontaneous activity of the subplate neurons becomes involved in modifying spontaneous movements (Milh et al. 2007). After birth, the spontaneous movements, such as general movements, are maintained as the major body movements (Prechtl 2001). The characteristics of general movements change at approximately 2 months after birth: from writhing to fidgety movements, and gradually disappear after 3 months (Einspieler and Prechtl 2005). This process coincides with the timing at which the subplate neurons undergo degeneration and the cortical neurons play major roles in movement generation, suggesting that the subplates play an important role in the regulation of spontaneous movements until 2 months of age (Hadders-Algra 2007, 2018). In addition, the thalamocortical pathway for transmitting sensory information to the cortex is first formed in the subplate, and the subplate neurons respond to sensory stimuli (Molnár et al. 2020). Therefore, it is speculated that the basis for the reinforcement of spontaneous movements in response to sensory stimulation can already be in place in the later fetal period, and that the behavior of the mobile task in 2-month-old infants may be generated by a mechanism similar to that established during the fetal period.

At 3 months of age, the subplates are already degenerated, and the cerebral cortex is thought to exhibit mature function. At this age, general movements start to diminish and goal-directed movements emerge from exploiting a specific pattern of spontaneous movements favorable to explore the physical world and from suppressing unnecessary spontaneous movements (Watanabe and Taga 2006; Hadders-Algra 2007; Watanabe et al. 2011). To generate such movements, one needs to have cortical mechanisms to create multiple stable states and make state transitions according to the situation and goal (Kelso 1995; 2012). In this model, the control parameter *b* causes bifurcation between the monostable and bistable states in the equation of the variable *v* representing the function of the cerebral cortex. The two equilibrium points posterior to the pitchfork bifurcation, with increases in the parameter *b*, have larger positive and negative values, amplifying the difference between the enhancement and inhibition of action, respectively.

The bifurcation diagram of *u* infers that there exists an intermediate stage between the model of 2-month-old (*b* = − 5) and the model of 3-month-old (*b* = 15) in the regime with *b* = 0 to 12, where the model showed the presence of bistable states but no phase transition to the further enhanced state. Our model predicts that the developmental process of action differentiation may undergo three stages.

The parameter *b* also represents the inter-individual differences of sensitivity to contingency. In the previous experimental research, the learning curves were calculated as a group average over the age. It is possible that the manipulation of averaging may dismiss the detailed changes. The parameter estimation of experimental data is a powerful tool to tackle this problem. Estimating parameters like *b* of each infant’s data will provide detailed information on the developmental process of action differentiation.

In addition, brain imaging studies have revealed that the cortical mechanism involved in audiovisual changes rapidly develops between 2 and 3 months, enabling the mature processing of stimulus features (Watanabe et al. 2008, 2010). These cortical mechanisms are thought to be essential for contingency detection in mobile tasks using signals of self-movement involved in the movement generation and sensory signals triggered by the mobile movements. This has been supported by experiments measuring the event-related potentials of the brains of 3-month-old infants in response to visual stimuli, in which the contingencies between self-generated movements and visual stimuli were manipulated (Zaadnoordijk et al 2020; Meyer and Hunnius 2021).

In summary, our model showed that the behavioral development in MCR can be understood by the variation of control parameter *b*, which changes on the timescale of weeks or months and reflects age-dependent and/or individual differences in the degree of subplate degeneration and cortical maturation.

### Developmental emergence of physical agency

Finally, we discuss how physical agency (Gergely 2002) emerges in early infancy. It has been suggested that experiments with young infants using the MCR paradigm can provide clues to understanding the manifestation of physical agency (Watanabe et al. 2011; Sen and Gredebäck 2021; Bednarski et al. 2022). Kelso and Fuchs (2016) constructed a dynamical system model including a mechanism of positive feedback, in which a mobile movement that accompanied a movement further enhanced it. In that model, increasing the control parameter caused a transition in the phase relationship between the limb oscillations and mobile oscillations, and stabilized the movements in the highly enhanced state. They further argued that this transition was the origin of agency.

In our model, it was revealed that increasing the value of the control parameter caused a similar phase transition from a state in which the mobile was passively pulled by the oscillatory movements of the limb to a state in which the mobile and limb oscillations mutually enhanced each other under the interaction condition. The latter state converged to a stable attractor as a whole system through rhythmic interactions of the brain, body and environment; this phenomenon has been referred to as “global entrainment” (Taga et al. 1991; Taga 2021). This is consistent with Gibson’s view emphasizing the bidirectional coupling of action and perception (Gibson 1977) and is considered to represent an important requirement for adaptive behavior to occur in the environment. Contingency detection between the signals of self-movements and environmental events was required for the emergence of the adaptive behavior from spontaneous movements, which do not necessarily rely on the absolute representation of self-movement but on the circular interaction between the infant and the environment. Furthermore, this study clarified that this phase transition did not occur under stimulation conditions. In other words, the transition occurred only when the limb oscillation and mobile oscillation had bidirectional interactions. This abrupt change, which may represent an important property for the generation of behavior as a physical agency, is produced by the nonlinear interaction between the infant and the environment and cannot be accounted for by a simple reinforcement learning model in which the probability of action is asymptotically changed (Zaadnoordijk et al 2018).

However, to act as a physical agency, one must change their behavior in the timing as they want, which further requires a meta-system to enable the choices of whether to act. In this sense, not only the enhancement of action, but also the inhibition of action according to the situation infers the mechanism for physical agency (Watanabe et al. 2011). Thus, in addition to the positive feedback mechanism of the KF model, the present model incorporated the dynamics of multiple internal states for changing behaviors according to the situation. The model also incorporated a mechanism for correlating signals originated from mobile movements and signals of self-movement, so as to be sensitive to precise differences in contingencies between the infant's own actions and environmental changes. As a result, the model of this study showed the capability to inhibit the action and thereby break the specific action-perception coupling and create a new mode of action-perception coupling. This study further captured the developmental changes in action differentiation between 2-month and 3-month-old infants as a bifurcation phenomenon. This provides a framework for understanding the mechanisms of the development of physical agency.

## Supplementary Information

Below is the link to the electronic supplementary material.

Supplementary file1 (DOCX 13 KB)

Supplementary file2 (MP4 3324 KB)

Supplementary file3 (MP4 18640 KB)

Supplementary file4 (MP4 17757 KB)

Supplementary file5 (MP4 27363 KB)

Supplementary file6 (MP4 17601 KB)
