# Supplementary material for: Dynamical systems model of development of the action differentiation in early infancy: a requisite of physical agency
Source: Biol Cybern. 2023 Jan 19;117(1-2):81–93. doi: 10.1007/s00422-023-00955-y (PMC10160167; doi:10.1007/s00422-023-00955-y)
Supplement: Supplementary file 1 — Supplementary file1 (DOCX 13 KB) [file 422_2023_955_MOESM1_ESM.docx]

Caption of Supplementary Videos

All of the video files contain the animation of the limb, the mobile, and the changes in *u*(t) and *v*(t). Simulated displacements of *x*(t) and *y*(t) are illustrated as those of the limb and mobile in the x-axis, respectively. In the interaction condition, the grey line between the limb and the mobile represents the ribbon in mobile conjugate reinforcement.

Video 1 : Baseline (c = 0, and *f* = 0).

Video 2 : Interaction condition of 2-month-old (*b* = –5, c = 2, and *f* = 0).

Video 3 : Stimulation condition of 2-month-old (*b* = –5, c = 0, and *f* = 2sin(2πt/6)).

Video 4 : Interaction condition of 3-month-old (*b* = 15, c = 2, and *f* = 0).

Video 5 : Stimulation condition of 3-month-old (*b* = 15, c = 0, and *f* = 2sin(2πt/6)).
